# Silent Migration for Three Decades, Intravesical and Sigmoid Colon Perforation by a Forgotten IUCD: A Rare Case Report

**DOI:** 10.1002/ccr3.73157

**Published:** 2026-07-26

**Authors:** Ayesha Ahmad, Iftikhar Khan, Muhammad Hassaan Javaid, Hira Habib, Abubakr Mahmoud

**Affiliations:** ^1^ Khyber Medical University Peshawar Pakistan; ^2^ FMH College of Medicine and Dentistry Lahore Pakistan; ^3^ Shifa College of Medicine Islamabad Pakistan; ^4^ Allama Iqbal Medical College Lahore Pakistan; ^5^ Faculty of Medicine University of Khartoum Khartoum Sudan

**Keywords:** bladder perforation, calcified intravesical mass, IUCD complications, laparoscopic removal, uterine perforation

## Abstract

IUCD migration can cause severe complications like bladder and sigmoid perforation, even decades post‐insertion. Regular follow‐up, timely imaging, and thorough patient education are critical for early detection and multidisciplinary management to prevent long‐term adverse outcomes and ensure patient safety in reproductive health care.

## 1 Introduction

Intrauterine contraceptives (IUCD) are preferred for cost effective long term reversible contraception (0.2% failure rate for levonorgestrel IUCDs and 0.8% for copper IUCDs) [1]. Nevertheless, rare though they are, some complications, such as uterine perforation (0.3–2.6 per 1000 insertions), may result in migration to adjacent organs, such as the sigmoid colon (common) and the bladder (rare) [2, 3]. Uterine perforation may be classified as partial (penetration into the myometrium) or complete (penetration into the peritoneal cavity) [4, 5]. Risk factors such as postpartum insertion, lactation and history of pelvic surgery have been mentioned [6]. The preferred imaging modality is ultrasonography, followed by X‐ray or CT for exact localization [7]. Retrieval is preferably performed by a minimally invasive approach (laparoscopy) [8].

Simultaneous perforation of the urinary bladder and bowel by a migrated IUCD is exceptionally rare. Most extrauterine IUCDs are single‐organ involvement, while combined bladder and bowel involvement has been described only in a few case reports worldwide [17, 18, 19, 20]. Although retained or migrated IUCDs have been reported after long asymptomatic periods, delays > 3 decades are extremely rare [16, 17, 18]. Our case is hence one of the longest reported intervals between IUCD insertion and symptomatic combined bladder–sigmoid colon perforation, underscoring the possibility of silent migration over decades [17, 18, 19, 20].

This paper describes a 32 year post‐insertion IUCD migration, resulting in bladder and sigmoid perforation. The reporting of this work follows the CARE guidelines for case reports [9].

## 2 Case Presentation

A 55‐year‐old multiparous woman had undergone insertion of an intrauterine contraceptive device (IUCD) approximately 32 years before presentation. The exact IUCD type could not be determined because of unavailable medical records. Following insertion, she did not attend routine gynecological follow‐up visits, and the device was presumed to have remained in situ. Two years before presentation, she underwent a total hysterectomy for benign gynecological disease. The IUCD was neither visualized nor removed during the procedure, suggesting that extrauterine migration had already occurred before hysterectomy. Because the device had likely perforated the uterus years before surgery and migrated into the pelvis, it was no longer located within the uterine cavity and therefore was not identified during hysterectomy. Several weeks before presentation, she developed progressive burning micturition, urinary frequency, hematuria with pyuria, and bilateral flank pain, prompting evaluation.

Physical examination revealed left flank tenderness, a soft abdomen, and normal post‐hysterectomy genitalia. Vitals were: temperature 100.2°F, blood pressure 132/85 mmHg, heart rate 88 bpm, respiratory rate 18/min. Initial differential diagnoses included complicated urinary tract infection (UTI), pyelonephritis, vesical stones, or IUCD migration.

### 2.1 Investigations

**FIGURE 1:**
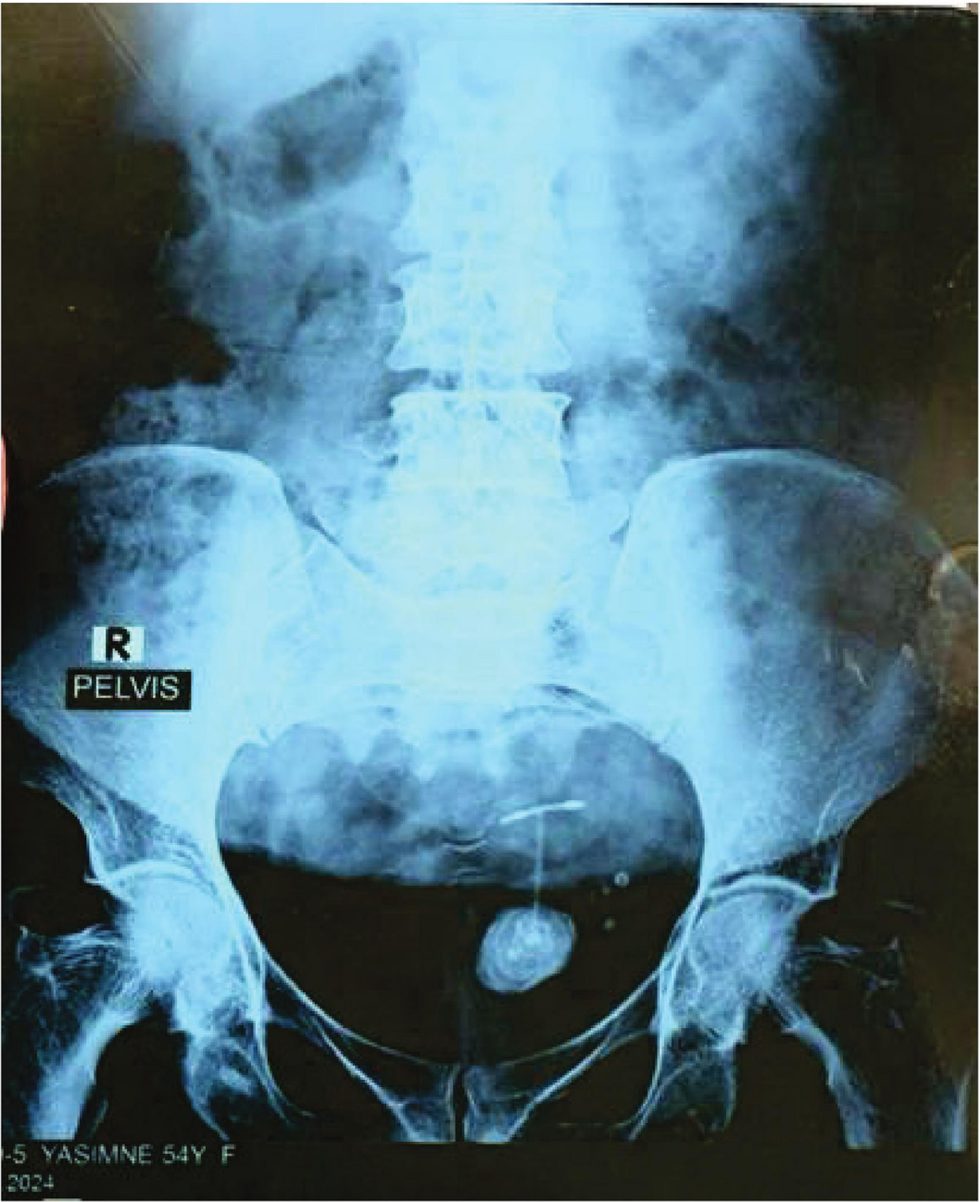
Abdominal and Pelvic X‐ray: Identified a radiopaque foreign body suggestive of an IUCD outside its normal position.

**FIGURE 2:**
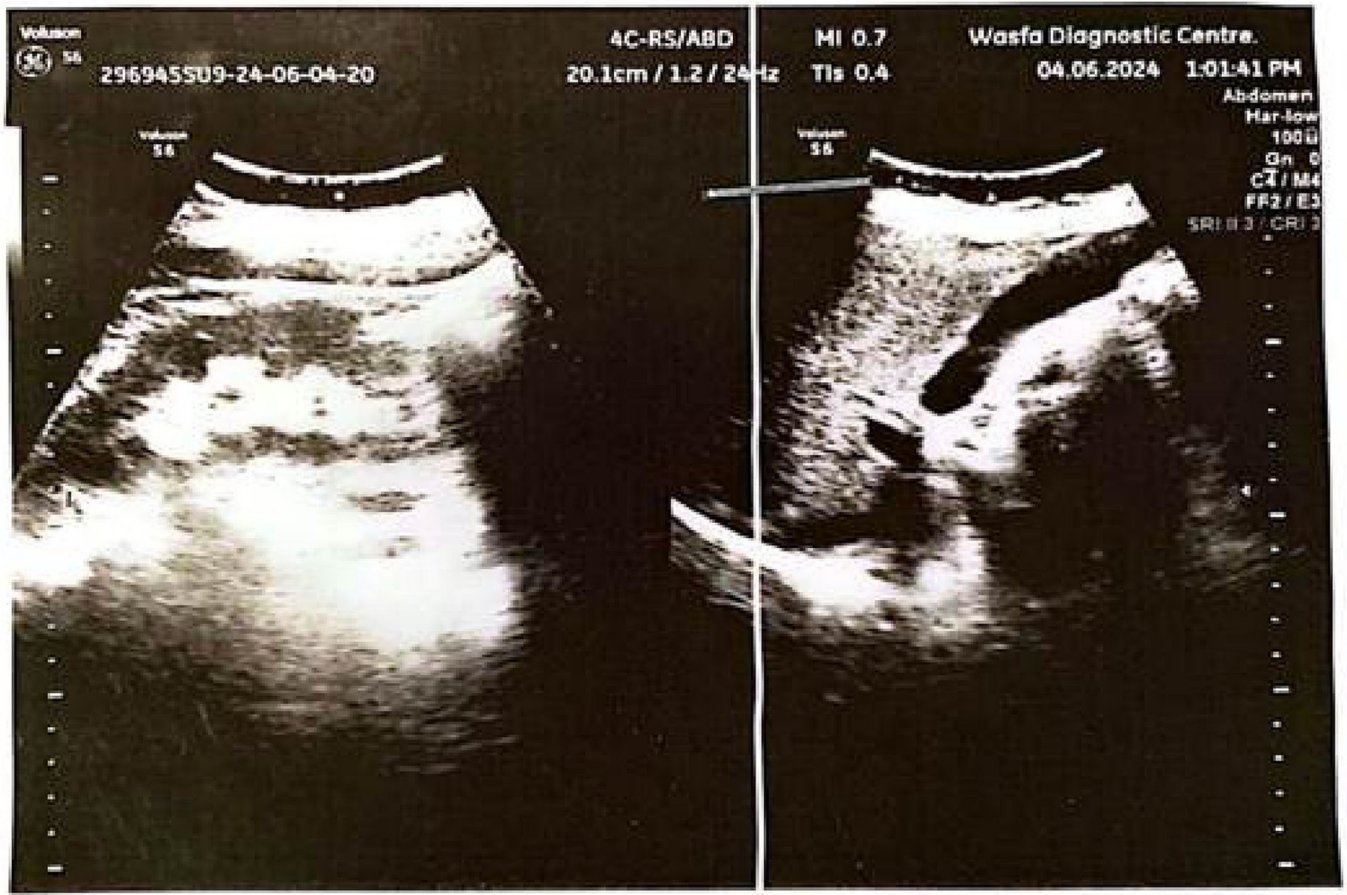
Ultrasound of Abdomen and Pelvis: Showed a calcified lesion at the bladder base with associated bladder wall thickening.

Ultrasound findings of the abdomen raised suspicion about the presence of vesical calculus. Contrast‐enhanced CT of the abdomen and pelvis revealed a T‐shaped hyperdense foreign body consistent with a migrated IUCD. One limb traversed the posterior wall of the urinary bladder, acting as a nidus for a densely calcified intravesical calculus while the opposite limb extended through the adjacent sigmoid colon wall without evidence of free intraperitoneal air or generalized peritonitis. Associated circumferential bladder wall thickening and surrounding inflammatory changes confirmed chronic transmural migration. CT showed an intravesical calculus 3.2 × 2.7 cm surrounding the intravesical limb of the IUCD (Figures 1 and 2).

Urinalysis showed cloudy urine (pH 6.0), positive leukocyte esterase and nitrites, trace protein, significant blood, high white blood cells (WBCs), red blood cells (RBCs), and bacteria. Urine culture confirmed *Escherichia coli* (> 100,000 CFU/mL). Complete blood count revealed elevated WBCs (14,000/μL).

### 2.2 Diagnosis Assessment

Migrated IUCD with perforation of the bladder and sigmoid colon, secondary to prior uterine perforation, with secondary bladder stone formation and complicated UTI. The IUCD was located partially within the urinary bladder and partially embedded in the sigmoid colon after complete uterine perforation.

### 2.3 Management

A multidisciplinary team (urology, gynecology, general surgery) initiated IV antibiotics (piperacillin‐tazobactam) and pain management (NSAIDs, acetaminophen). Surgery involved:

1. Endoscopic cystolitholapaxy to remove bladder calculi.
2. Diagnostic laparoscopy confirming IUCD perforation.
3. Laparoscopic IUCD removal, bladder repair with absorbable sutures, and sigmoid colon repair without bowel resection.

IV metronidazole and ceftriaxone continued for 5 days, with a urinary catheter maintained for 5 days. The patient was educated on surgical risks (e.g., persistent UTI, fistula formation) and follow‐up importance. Monitoring included checks for infection, bleeding, and bowel obstruction.

### 2.4 Follow Up and Outcomes

The postoperative course was uneventful. Her urinary catheter was removed on postoperative day (POD) 7 after confirming satisfactory bladder healing. She had complete resolution of her dysuria, frequency, hematuria, and flank pain. She had normal bowel habits without constipation, abdominal pain, or fecal leak or symptoms consistent with an enterovesical fistula. Her repeat urine culture 2 weeks later was sterile, and her renal function tests remained normal. Four weeks after surgery, ultrasonography revealed complete bladder healing without residual calculi, leakage, hydronephrosis, or pelvic collection. Three and 6 months after the procedure, she remained asymptomatic with preserved urinary and bowel function and no radiologic evidence of recurrent disease.

## 3 Discussion

IUCD migration, often due to uterine perforation at insertion or gradual erosion via pressure necrosis, is rare but serious [3, 5]. The primary perforation may occur during insertion by mechanical forces, with known risk factors including inadequate training of family planning providers and insertion at early puerperal period [10]. The symptoms of an IUD perforation are diverse, varying from a subsequent unwanted pregnancy to irritant lower urinary tract symptoms, chronic pelvic pain, peritonitis, and fistulae or abscess formation depending on the organ of penetration and the interval since penetration and the patient's response [11, 12]. In a small number of cases, bladder perforation by IUCD has been associated with intravesical stone formation [13, 14].

Although intravesical migration of IUCDs with subsequent bladder stone formation has increased, simultaneous bladder and sigmoid colon perforation remains extremely rare. Although most cases report migration into a single pelvic organ, combined bladder–bowel involvement has only been reported sparingly. Our case thus provides valuable evidence for the natural history and surgical management of delayed multiorgan migration.

Delayed presentation after IUCD migration has been reported in isolated cases over several years up to multiple decades. A retained Lippes Loop was found after approximately 50 years, and several migrated IUCDs were asymptomatic for almost 30 years before diagnosis [16, 17, 18]. In comparison with previously published reports of bladder and bowel perforation presenting after 10–20 years [19, 20, 21, 22], our patient's presentation 32 years after insertion represents one of the longest times associated with simultaneous bladder and sigmoid colon perforation (Table 1).

**TABLE 1:** Risk factors contributing to IUCD complications, modified from [6] to highlight factors relevant to this case.

Risk factor	Mechanism	Clinical implications
Postpartum insertion	Softer uterine wall increases perforation risk	Early migration risk
Cesarean delivery	Scar tissue alters IUCD placement	Embedding in scar or adjacent organs
Lactation	Uterine atrophy weakens wall	Increased perforation potential
Previous pelvic surgery	Adhesions alter anatomy	Complicates retrieval, involves bowel/bladder
Improper insertion	Excessive force or misplacement	Immediate or delayed perforation
Delayed diagnosis	Unnoticed migration	Long‐term complications (e.g., stones, infections)

Imaging was crucial, with CT identifying the IUCD's ectopic location following initial misdiagnosis as a vesical stone [11, 23]. The bladder calculi were caused by chronic irritation from the IUCD, which was compatible with previous studies [13, 14, 24, 31]. The hybrid endoscopic‐laparoscopic approach limited invasiveness, consistent with literature that encourages minimally invasive retrieval [25, 27]. The World Health Organization recommends removal of a dislocated IUD as soon as possible irrespective of their type and location, and it is advised to retrieve a migrated IUD by minimally invasive techniques [26]. The histopathological changes listed (hyaline, cystic, fatty, mucoid degeneration) are real leiomyoma pathology findings, but the qualifier “with intramural leiomyoma impacted IUD cases” cannot be supported by any readily identifiable primary source [28]. A review of surgical techniques to remove IUD revealed that 93% of the reported cases in literature attempted laparoscopically, but cases of both abdominal and pelvic organ perforations have a conversion to open surgery rate of 22.5% [14, 15, 29, 32].

Her previous hysterectomy likely delayed recognition as the IUCD had already migrated out of the uterine cavity prior to surgery and thus wasn't identified intraoperatively. Thus, a missing IUCD should always lead to radiologic localisation pre‐ or post‐pelvic surgery.

Recent guidelines recommend adherence to proper insertion protocols and timing to minimize perforation risk [2, 30]. Post‐operative care and follow‐up averted the complications, with satisfactory results at 4 weeks.

This emphasizes the importance of routine follow‐up of IUCD and patient education to seek treatment early. IUCD migration must be kept in mind in patients presenting with urinary or GI symptoms even many years after insertion, and advanced imaging should be used to diagnose it.

## 4 Conclusion

IUCD migration with multi‐organ perforation is a rare but manageable complication. This case highlights the importance of regular follow‐ups, timely imaging, and multidisciplinary surgery to achieve favorable outcomes. This case reminds us that we must never consider a missing IUCD “expelled” unless we are certain radiologically. Decades after insertion, migrated devices can cause bizarre multiorgan complications requiring multidisciplinary minimally invasive management.

## Author Contributions

**Ayesha Ahmad:** conceptualization, methodology, software. **Iftikhar Khan:** software, methodology, conceptualization. **Muhammad Hassaan Javaid:** conceptualization, formal analysis, supervision, visualization, project administration. **Hira Habib:** conceptualization, project administration, writing – original draft, writing – review and editing, resources. **Abubakr Mahmoud:** writing – original draft, writing – review and editing.

## Funding

The authors have nothing to report.

## Ethics Statement

The authors have nothing to report.

## Consent

Written Informed consent was taken from the Patient for the publication.

## Conflicts of Interest

The authors declare no conflicts of interest.

## Data Availability

Data sharing does not apply to this article as no datasets were generated or analyzed during the current study.
